# The cure model in perinatal epidemiology

**DOI:** 10.1177/0962280220904092

**Published:** 2020-02-11

**Authors:** Emil A Stoltenberg, Hedvig ME Nordeng, Eivind Ystrom, Sven O Samuelsen

**Affiliations:** 1 Department of Mathematics, University of Oslo, Oslo, Norway; 2 PharmaTox Strategic Research Initiative, Faculty of Mathematics and Natural Sciences, University of Oslo, Oslo, Norway; 3 PharmacoEpidemiology and Drug Safety Research Group, University of Oslo, Oslo, Norway; 4 Norwegian Institute of Public Health, Oslo, Norway; 5 Department of Psychology, University of Oslo, Oslo, Norway

**Keywords:** Perinatal epidemiology, paracetamol, attention-deficit hyperactivity disorder, frailty model, Cox regression, logistic regression, censoring, mother–child studies

## Abstract

In the statistical literature, the class of survival analysis models known as cure models has received much attention in recent years. Cure models seem not, however, to be part of the statistical toolbox of perinatal epidemiologists. In this paper, we demonstrate that in perinatal epidemiological studies where one investigates the relation between a gestational exposure and a condition that can only be ascertained after several years, cure models may provide the correct statistical framework. The reason for this is that the hypotheses being tested often concern an unobservable outcome that, in view of the hypothesis, should be thought of as occurring at birth, even though it is only detectable much later in life. The outcome of interest can therefore be viewed as a censored binary variable. We illustrate our argument with a simple cure model analysis of the possible relation between gestational exposure to paracetamol and attention-deficit hyperactivity disorder, using data from the Norwegian Mother, Father and Child Cohort Study conducted by the Norwegian Institute of Public Health, and information about the attention-deficit hyperactivity disorder diagnoses obtained from the Norwegian Patient Registry.

## 1 Introduction

Perinatal epidemiological studies investigating the possible effects of some gestational exposure on a postnatal condition can roughly be split into two categories. Those where the condition is observable immediately after birth and those where it may take years before the condition is ascertained, if ever. This paper is concerned with the latter. Smoking and low birth weight; infant supine position and sudden infant death syndrome; and foetal alcohol spectrum disorders fall in the first category. The association between prenatal marijuana exposure on neuropsychological conditions^1^ and the association between prenatal exposure to pharmaceuticals and neurodevelopmental disorders belong to the second category. The present study was motivated by the hypothesis linking gestational exposure to paracetamol and an increased risk of neurodevelopmental disorders, attention-deficit hyperactivity disorder (ADHD) in particular,^2–5^ hypotheses that are pertinent examples of the latter category.

From a statistical modelling perspective, the main difference between these two types of hypotheses is that the data in the latter are plagued by censoring. That is, the outcome in studies in the second category may be unknown at the time of study due to a lack of follow-up. Thus, for studies in the first category, standard regression analysis is a natural choice (e.g. linear, Poisson, logistic), while for the latter type of studies, the need to handle censoring often leads to survival analysis methods being employed (e.g. the Cox model). A consequence of opting for a survival analysis model is that the outcome is defined as the *time to diagnosis*, a convenient choice due to the availability of efficient survival analysis software, but that, we argue, can in many cases be an imprecise operationalisation of the outcome in view of the hypothesis being tested. The reason for this is that in perinatal studies belonging to our second group, the hypotheses often concern an exposure that is only present during pregnancy, and consequently the outcome of interest should be thought of as occurring when the effect of the exposure ceases to have an effect, that is, at birth. Think of a frailty model with hazard Zα(t), where *Z* is a frailty variable. Our reading of the hypotheses in the second group is that they concern the effect of the exposure on the distribution of *Z*, but not on α(t). By defining the outcome as the time to diagnosis, one is effectively testing another hypothesis than initially intended. In this paper, we show that in cases where the outcome (occurring at birth, but unobservable at that time) can be thought of as binary, the class of statistical models known as cure models is a viable alternative to standard regression and survival analysis models. In concluding, we also propose modelling alternatives for situations where the unobservable outcome variable is continuous, and for situations where the presence of the condition under study can be ruled out during the course of a life.

In the statistical literature, cure models have received much attention in recent years.^6–12^ The name stems from medical applications where some patients never experience a relapse of the disease under study, and these patients are therefore considered cured. Cure models have also been proposed in the field of reproductive epidemiology to account for the possibility of some of the individuals under study being sterile.^13^

It is worth noting that the motivation typically underlying cure models is rather different from the argument we put forward in this paper. Typically, cure models are solidly anchored in the survival analysis world, while our approach, which is focused on the probability of belonging to the susceptible group, is more akin to a misclassification- or missing data problem. In other words, in this paper, we are less interested in survival quantities such as hazard rates and survival functions per se, but view them as nuisance parameters that must be tended to in order to make inferences on the parameters determining whether a child is born susceptible or not. See Farewell^14^ for an early paper advocating for cure models in a similar manner.

The article proceeds as follows. In Section 2, we provide a brief introduction to the cure model, and motivate this class of models in light of the hypothesis linking paracetamol and ADHD (hereafter referred to as the paracetamol–ADHD hypothesis). This section also contains some theoretical results on simple logistic and Cox models when such are fitted to data that contain a cure fraction. These results are illustrated with two small simulation studies. In Section 3, we fit different cure models to the data on gestational exposure to paracetamol and ADHD, and compare these with a logistic regression and a Cox regression model. The aim of this application is to investigate whether our reading of the paracetamol–ADHD hypothesis finds empirical backing, and illustrate the fact that all three classes of models are likely to lead to rather similar conclusions about the paracetamol–ADHD hypothesis.

## 2 The cure model and ADHD

In this section, we first, using the paracetamol–ADHD hypothesis as our example, elaborate on why we find the class of cure models appropriate for the perinatal studies discussed in this paper. Subsequently, we give a brief introduction to the standard mixture cure model.

### 2.1 The paracetamol–ADHD hypothesis

**Figure 1.**
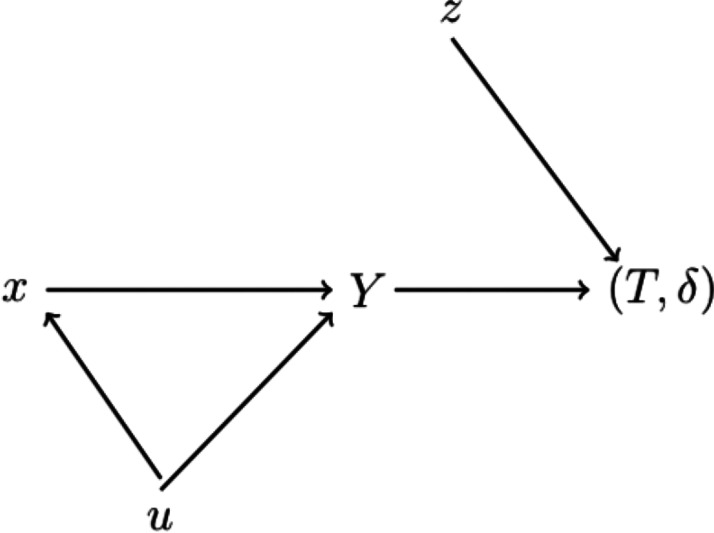
A DAG illustrating the data generating mechanism presented in Section 2.1. The exposure of interest (paracetamol) is *x*, *Y* is the latent susceptible/nonsusceptible indicator and *u* is a confounder of this relation. Given susceptibility (*Y *=* *1), *z* is a postnatal covariate influencing the possibly right-censored time to diagnosis (T,δ).

The use of cure models in perinatal epidemiological studies can be motivated by the directed acyclic graph (DAG) in Figure 1. In this DAG, *x* represents the gestational exposure, *Y* is a binary indicator representing the condition the child is born in, while *u* is a set of confounders. In perinatal studies belonging to our second category, we think of *Y* as an indicator of a being born susceptible (*Y *=* *1) or nonsusceptible (*Y *=* *0) to the condition in question, i.e. the variable *Y* indicates the incidence of, or vulnerability to, a particular disease or condition, or a lifetime free of the disease or condition under study.^14^ The variable *T* is the minimum of the time at which the presence of the condition in the child is discovered and a censoring time, *δ* is an indicator taking the value 1 if the value *Y *=* *1 is discovered before censoring and *z* is a set of postnatal variables influencing the time to an eventual diagnosis. The paracetamol–ADHD hypothesis suggests that gestational exposure to paracetamol is associated with ADHD. More precisely, it states that – all else equal during the gestational period – two children with the exact same gestational exposure to paracetamol should lead to the same conclusion about the effect of gestational exposure to paracetamol on the risk of ADHD, *even though the two children were diagnosed at different ages*. This entails that the exposure effectively ceases to have an effect once the child is born, which is the reason for there not being a direct arrow from *x* to (T,δ) in Figure 1. From this perspective, the outcome variable of interest is not the time to diagnosis, nor is it the time to onset of ADHD, but rather a latent susceptibility variable whose realisation takes place when the exposure ceases, which is at birth. This latent variable is represented by the *Y* in the DAG, so according to the hypothesis, it is the relation between *x* and *Y* we seek to make inferences on. That is, had *Y* been observable, we would have analysed the relationship between *x* and *Y* by a binary regression analysis.

The *Y*’s are, however, only partially observable so the T,δ's are what we have at our disposal for making inferences on the relation between *x* and *Y*. It is tempting to use the censoring indicators *δ* as stand-ins for the latent *Y*’s. The problem with this is that the probability of observing a diagnosis is not the same as the probability of being susceptible. The former probability depends on the distribution of the diagnosis times, hence the need to model the diagnosis times, which is what the cure model of the next section does.

### 2.2 The standard cure model

As above, let *Y* be the indicator of susceptibility (*Y *=* *1), or of a lifetime free of the condition (*Y *=* *0), with *π* the probability of *Y *=* *1. The time to diagnosis is a variable $\tilde{T}$ subject to right censoring, i.e. what we observe is $T=\min\left\{\tilde{T},C\right\}$ and $\delta =I\{\tilde{T}\leq C\}$, where *C* is a random censoring time. The standard cure model takes the population survival function as given by

(1) $$S_{\text{pop}}(t)=1-\pi +\pi \;S(t),$$

where $S(t)=\mathrm{Pr}\left(\tilde{T}\geq t|\text{susceptible}\right)$ is the survival function of the susceptible group. This latter survival function is assumed to be proper in the sense that it tends to 0 as t→∞, hence $S_{\text{pop}}(t)\to 1-\pi$, which is the nonsusceptible fraction of the population.

Both *π* and *S*(*t*) are typically modelled as functions of covariates, common choices being a logistic function for the probability of being susceptible, and a proportional hazards model for the survival function of the susceptible group. That is, for the *i*’th individual

(2) $$\pi_{i}=\pi \left(x_{i}^{\text{t}}\beta \right)=1/\left(1+\exp\left(-x_{i}^{\text{t}}\beta \right)\right),\;\text{ and }\;S_{i}(t)=\exp\{-\exp(z_{i}^{\text{t}}\gamma )\int_{0}^{t}\alpha_{0}(s)\;\text{d}s\},$$

in terms of a baseline hazard function $\alpha_{0}(t)$ that might be parametric or nonparametric, and covariate vectors *x_i_* and *z_i_* that can be equal, overlapping or completely different. As regards perinatal studies, an important feature of the cure model (equation (2)) is that it allows the researcher to distinguish between prenatal and postnatal covariate effects. The covariate vector *x* governs the distribution of *Y*, while the covariate vector *z* governs the distribution of the diagnosis times. This means that in order to give the effect estimates of the covariates in *x* a direct causal meaning, they must be present during the gestational period. The covariate vector *z*, on the other hand, might contain covariates that do not influence the foetus, such as characteristics of the kindergarten or the school the child attends.

For the perinatal studies that are the object of this paper, two features of the model in equation (1) should be pointed out. First, by using this model, we are assuming that the nonsusceptible individuals are never diagnosed with the condition in question, that is, we assume that there are no false positives in the sample. In the case of ADHD, this assumption may be questioned. In the US, there is evidence of ADHD overdiagnosis in some communities,^15^ meaning that the prevalence of ADHD is higher than the standard 3–5% prevalence estimate.^16–18^ In the data set we analyse in Section 3, only about 2.3% of the children are diagnosed with ADHD. Since this number is well below the standard prevalence estimates, it would lead one to believe that false positives are not a major issue in our data.

Notice that if *δ* = 1, then we know that *Y *=* *1, while if *δ* = 0, we do not know whether the individual is susceptible or nonsusceptible. This brings us to the second point, if the data contain information on nonsusceptibility (e.g. a medical test that ascertains immunity to a certain disease), then this information ought to be taken into account. As it stands, the model in equation (1) cannot incorporate such information (see Remark 1 in Section 4 for further discussion).

The log-likelihood function of the model in equation (2) is

ℓn(β,γ,α0)=∑i=1n{δi(log⁡ πi+log⁡ α0(ti)+zitγ+log⁡ Si(ti))+(1−δi)log⁡(1−πi+πiSi(ti))}.

If $\alpha_{0}(t)$ is parametrically specified, it is straight forward to maximise this log-likelihood. When the hazard rate is nonparametric, the log-likelihood can be maximised using the expectation-maximisation algorithm introduced in Sy and Taylor^7^ and Peng and Dear.^8^ The R-package smcure^19^ implements this algorithm. The asymptotic theory of the maximum likelihood estimator in the semiparametric case was worked out by Fang et al.^9^ and Lu^10^, building on previous work of Murphy^20^ for the Gamma frailty model.

### 2.3 Fitting logistic and Cox models to cure data

In this section, we provide some insight on the bias incurred in the parameter estimates when the data stem from a cure model, but a logistic regression model or a Cox regression model, is chosen.

Suppose that the data (T,δ) are generated from a model with survival function

(3) $$S_{\text{pop}}\left(t\right)=1-\pi \left(\beta_{0}+\beta_{1}x\right)+\pi \left(\beta_{0}+\beta_{1}x\right)S(t),$$

where π(u)=exp⁡(u)/(1+exp⁡(u)), *x* a binary indicator and *S*(*t*) is a proper survival function that can be expressed as S(t)=exp⁡(−A(t)), in term of the cumulative hazard *A*(*t*). Thus, we assume that the true data generating mechanism is that of a cure model. Our parameter of interest is *β*_1_, giving the effect of the exposure *x* on susceptibility (*Y *=* *1).

Consider fitting a logistic regression model to independent data $\left(T_{1},\delta_{1}),\ldots ,(T_{n},\delta_{n}\right)$ generated by equation (3), with fixed covariates $x_{1},\ldots ,x_{n}$, and independent censoring. The expectation of *δ* given *x* is $\text{E}\;[\delta |x]=\text{E}\;\text{E}\;[I\{\tilde{T}\leq C\}|x,C]=\pi (\beta_{0}+\beta_{1}x){\text{E}}_{G}\;[1-S(C)]$, with ${\text{E}}_{G}[\cdot ]$ the expectation with respect to the distribution *G* of the censoring times. Since *x* is binary, we can define $n_{0}=\#\{i:x_{i}=0\},\;n_{1}=\#\{i:x_{i}=1\},\;\pi_{0}=\pi (\beta_{0})$ and $\pi_{1}=\pi (\beta_{0}+\beta_{1})$. The maximum likelihood estimators of *π*_0_ and *π*_1_ are

$${\hat{\pi }}_{0}=\frac{1}{n_{0}}\sum_{i:x_{i}=0}\delta_{i},\;\text{ and }\;{\hat{\pi }}_{1}=\frac{1}{n_{1}}\sum_{i:x_{i}=1}\delta_{i},$$

which converge in probability to E [δ | x=0] and E [δ | x=1], respectively. Being invariant under transformation, the maximum likelihood estimator of *β*_1_ is then

$${\hat{\beta }}_{1}=\log\frac{{\hat{\pi }}_{1}}{1-{\hat{\pi }}_{1}}-\log\frac{{\hat{\pi }}_{0}}{1-{\hat{\pi }}_{0}},$$

so that by continuous mapping

(4) $${\hat{\beta }}_{1}\overset{p}{\to }\log\frac{\pi_{1}{\text{E}}_{G}[1-S(C)]}{1-\pi_{1}{\text{E}}_{G}[1-S(C)]}-\log\frac{\pi_{0}{\text{E}}_{G}[1-S(C)]}{1-\pi_{0}{\text{E}}_{G}[1-S(C)]}=\beta_{1}-\log\frac{1+\exp(\beta_{0}+\beta_{1}){\text{E}}_{G}\;[S(C)]}{1+\exp(\beta_{0})\;{\text{E}}_{G}\;[S(C)]},$$

as *n* tends to infinity. From this expression, we see that the estimator ${\hat{\beta }}_{1}$ will be biased (negatively if $\beta_{1}>0$), and that the degree to which the estimator is biased depends on the distribution of the diagnosis times and on the value of *β*_0_ (and on the distribution of the censoring times).

If *S*(*t*) rapidly approaches zero, which is the case if the condition under study is likely to be discovered early in life, then the bias term will be small. And, through its dependence on *β*_0_, we see that the bias of ${\hat{\beta }}_{1}$ is less pronounced if the condition in question is rare, which is the case with ADHD (recall that only 2.3% of the children in our sample were diagnosed with ADHD).

Now, consider fitting a Cox regression model with hazard rate $h_{0}(t)\exp(\gamma x)$, with $h_{0}(t)$ left unspecified, to the data generated by equation (3). In this case, it turns out that if $\exp(\beta_{0}+\beta_{1}x_{i})$ is close to zero for all *x_i_*, then the point estimate $\hat{\gamma }$ obtained by maximising the Cox partial likelihood will not deviate much from the estimate ${\hat{\beta }}_{1}$ obtained by maximising the likelihood of the true model. The details are as follows (an excellent exposition of the machinery used in the following can be found in Gill^21^): the counting processes corresponding to the model in equation (3) are $N_{i}(t)=M_{i}(t)+\Lambda_{i}(t),\;i=1,\ldots ,n$, with

$$\Lambda_{i}(t)=\int_{0}^{t}Y_{i}(s)\pi \left\{\beta_{0}+\beta_{1}x_{i}-A(s)\right\}\;\text{d}A(s),$$

for i=1,…,n, where the $M_{i}(t)$ and $Y_{i}(t)$ are martingales and at-risk indicators, respectively; *A*(*t*) is the cumulative hazard of the susceptible individuals; while π(·) is the logistic function; and we have used that d log⁡Spop(t)=−π(β0+β1xi−A(t)) dA(t).

Let $B_{n}(t,\gamma )=\sum_{i=1}^{n}x_{i}Y_{i}(t)\exp(\gamma x_{i})/\sum_{i=1}^{n}Y_{i}(t)\exp(\gamma x_{i})$, then the score function $U_{n}(\gamma )$ of Cox’s partial likelihood is

$$U_{n}(\gamma )=\sum_{i=1}^{n}\int_{0}^{T}\{x_{i}-B_{n}(s,\gamma )\}\;\text{d}N_{i}(s)=\sum_{i=1}^{n}\left[\int_{0}^{T}\{x_{i}-B_{n}(s,\gamma )\}\;\text{d}M_{i}(s)+\int_{0}^{T}\{x_{i}-B_{n}(s,\gamma )\}\;\text{d}\Lambda_{i}(s)\right].$$

If the second term on the right is zero, which it is when the model $h_{0}(t)\exp(\gamma x)$ is the true model, then $U_{n}(\gamma )=0$ is an unbiased estimating equation. The function

$$\text{E}\;U_{n}(\gamma )=\text{E}\;\sum_{i=1}^{n}\int_{0}^{T}\{x_{i}-B_{n}(s,\gamma )\}\;\text{d}\Lambda_{i}(s)=\text{E}\;\sum_{i=1}^{n}\int_{0}^{T}\{x_{i}-\frac{\sum_{j=1}^{n}x_{j}Y_{j}(s)e^{\gamma x_{j}}}{\sum_{j=1}^{n}Y_{j}(s)e^{\gamma x_{j}}}\}Y_{i}(s)\frac{e^{\beta_{0}+\beta_{1}x_{i}-A(s)}}{1+e^{\beta_{0}+\beta_{1}x_{i}-A(s)}}\;\text{d}A(s),$$

is approximately zero when the function

$$x\;\mapsto \;g(x,t)=\exp(\beta_{0}-A(t))/\left\{1+\exp(\beta_{0}+\beta_{1}x-A(t))\right\},$$

is approximately constant. Since a more rapidly increasing cumulative hazard *A*(*t*) will on an average result in shorter lifetimes, the function x ↦ g(x,t) is approximately constant only when *β*_0_ is small, that is, if the probability of being susceptible to the event of interest is low.

In summary, when it comes to estimating *β*_1_, the logistic model provides decent estimates when *β*_0_ is small or the cumulative hazard increases rapidly, while the Cox model only gives decent estimates when *β*_0_ is small.

To illustrate this, we performed two simulations studies with varying parameter values. In both, the data were simulated from a cure model of the form given in equation (3), with the parameter of interest set to $\beta_{1}=1.5$, *x* being a binary exposure, the censoring variables were drawn from an exponential distribution with mean 8 and the sample size set to 4000.

**Figure 2.**
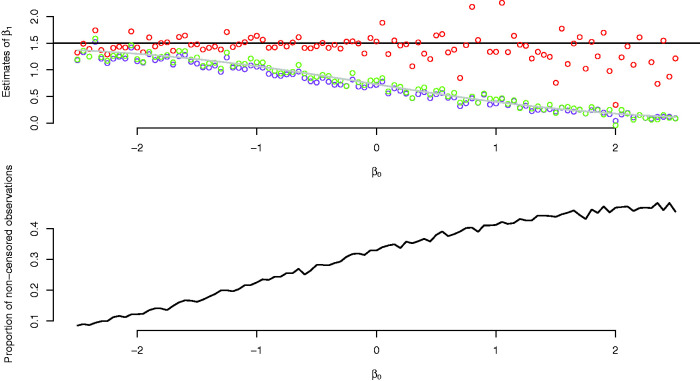
Upper panel: estimates of *β*_1_ from a semiparametric cure model (red dots), logistic model (green dots) and a Cox model (purple dots), with varying values of *β*_0_. The grey line overlapping the green and purple dots is the term on the right in equation (4) as a function of *β*_0_. The black line is the true parameter value of *β*_1_. Lower panel: Proportion of non-censored observations as a function of *β*_0_.

In the simulations reported in Figure 2, we set A(t)=t/8 (i.e. the lifetimes of the susceptible population stemmed from an exponential distribution with mean 8) and varied the *β*_0_ parameter. We see that the logistic model and the Cox model estimates are close to the truth for small values of *β*_0_, and that the bias of these estimators increases with *β*_0_. The increasing variability of the semiparametric estimates is due to $\pi (\beta_{0}+\beta_{1}x_{i})$ approaching one as *β*_0_ increases.

**Figure 3.**
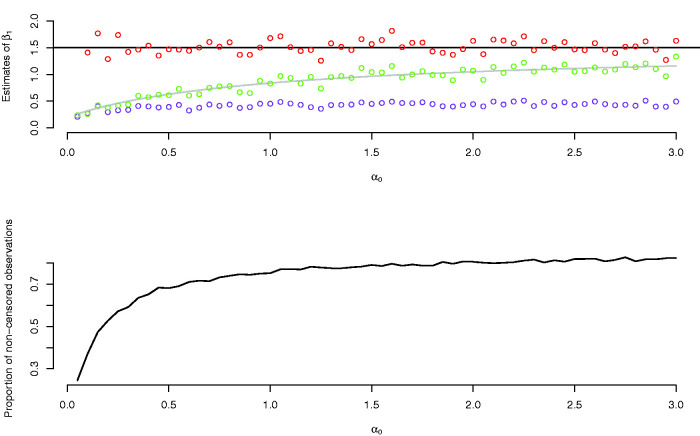
Upper panel: estimates of *β*_1_ from a semiparametric cure model (red dots), logistic model (green dots) and a Cox model (purple dots), with varying values of *α*_0_. The grey line is the term on the right in equation (4) as a function of *α*_0_. The black line is the true parameter value of *β*_1_. Lower panel: proportion of non-censored observations as a function of *α*_0_.

In the simulations reported in Figure 3, we set $\beta_{0}=1.2$ (thus $\pi (\beta_{0})=0.77$ and $\pi (\beta_{0}+\beta_{1})=0.94$) and varied the cumulative hazard A(t)=αt, taken to be that of exponential distributions. As the hazard rate *α* increases, the bias of the logistic model decreases, eventually converging to ‘unbiasedness’. Varying values of *α* does not, as discussed above, have an effect on the estimates of the Cox model.

In the data set we analyse in Section 3, only 2.3% of the children are diagnosed with ADHD. This indicates that *β*_0_ is small. Moreover, more than half of the diagnoses occur before the age of 12 years, indicating that the cumulative hazard increases quickly. The insights of the current section therefore suggest that we should expect to see a nominal similarity between the Cox model estimates and the estimates of the logistic model, as well as a similarity of both these estimates to the estimates of the logistic part of the cure models.

## 3 Data analysis

The cure models we fit to the paracetamol–ADHD data have population survival functions

(5) $$S_{\text{pop}}(t;x_{i},z_{i})=1-\pi (x_{i}^{\text{t}}\beta )+\pi (x_{i}^{\text{t}}\beta )S_{0}{\left(t\right)}^{\exp(z_{i}^{\text{t}}\gamma )},$$

with $\pi (x_{i}^{\text{t}}\beta )=\exp(x_{i}^{\text{t}}\beta )/\{1+\exp(x_{i}^{\text{t}}\beta )\}$ and $S_{0}(t)$ a baseline survival function being either nonparametric or that of a gamma distribution. The argument presented in Section 2.1 entails that we expect gestational exposure to paracetamol to have an effect on whether or not a child belongs to the susceptible group, but, given that a child belongs to the susceptible group, we do not expect paracetamol to have an effect on determining *when* in life the child might be diagnosed with ADHD. This means that if the exposure variable enters both covariates vectors in equation (5) (i.e. both *x_i_* and *z_i_*), then we anticipate that the true *β*- and *γ*-coefficients corresponding to the exposure should be positive and zero, respectively.

For comparison, we also fit a logistic model and a Cox model to the paracetamol–ADHD data. As elaborated on in Section 2.3, we have reason to expect a nominal similarity between the exposure estimates from these models to those of the corresponding cure models. This is because the prevalence of ADHD in the data is low, and because most children diagnosed with ADHD are diagnosed quite early in life.

The data used in this analysis stem from the Norwegian Mother, Father and Child Cohort Study (MoBa) conducted by the Norwegian Institute of Public Health. Information about the ADHD diagnoses was obtained from the Norwegian Patient Registry (NPR). The analyses of this section are motivated by and use essentially the same data as Ystrom et al.,^2^ and a more elaborate discussion of the MoBa and the NPR can be found therein.

After having removed observations with missing values, the sample consisted of n=95 545 units (pairs of mothers and one of their offspring). Among the children in this sample, 2 165 had been diagnosed with ADHD by the end of the follow up in 2016, that is about 2.3%, a number which is about half the international estimate of ADHD prevalence.^16–18^ The mean and median age at diagnosis were 10.8 and 11 years, respectively. Half of the children with a diagnosis of ADHD were diagnosed when they were between 9 and 12 years old, while the youngest and oldest child to be diagnosed were 1 and 16 years old, respectively. Table 1 gives the birth year of the children in the full sample (before deleting 7 713 observations due to missing values on the covariates), the number of diagnoses observed in the relevant birth cohort and the percentage of mothers who consumed paracetamol at least once during pregnancy in each cohort.

**Table 1.** The 11 birth year cohorts included in the data, size of cohort and number of children within each cohort with a diagnosis of ADHD.

Year	Births	Diagnosis	%	% Paracetamol
1999	46	0	0.00	41.3
2000	2010	89	4.43	38.7
2001	3950	137	3.47	41.3
2002	8331	338	4.06	41.8
2003	12,163	449	3.69	42.1
2004	13,085	398	3.04	43.2
2005	15,176	395	2.60	42.6
2006	16,858	278	1.65	42.8
2007	15,504	221	1.43	43.8
2008	12,910	78	0.60	42.8
2009	3225	5	0.16	44.1
Total	103,258	2388	2.31	42.7

Note: The last column is the percentage of mothers in the data who consumed paracetamol at least once during pregnancy.

The cure models we fit have population survival functions of the form (equation (5)), with the baseline survival function being either nonparametric or that of a gamma distribution with density $\left(b^{a}/\Gamma (a))t^{a-1}\exp(-bt\right)$. The four covariates we considered were binary indicators of gestational exposure to paracetamol; of whether paracetamol was consumed due to fever; of whether the mother consumed alcohol more than once a month during pregnancy; and of whether the mother had four years or more university education (or equivalents). The paracetamol indicator is the exposure of interest, while the three other covariates are potential confounders. Summary statistics for these covariates are presented in Table 2.

**Table 2.** Summary of covariates.

	ADHD (%)	not ADHD (%)	All (%)
Paracetamol	48.4	43.1	43.2
Mother educ.	40.4	65.2	64.6
Alcohol	0.5	0.2	0.2
Fever	9.6	7.5	7.6

ADHD: attention-deficit hyperactivity disorder.

Note: All the covariates are binary (0–1). For an individual, a value of 1 means, respectively, that paracetamol was consumed at least once during gestation, the mother has higher education, the mother consumed alcohol at least once a month during gestation and that paracetamol has been consumed to alleviate fever.

We fitted three different cure models for each of the two specifications of the baseline survival function $S_{0}(t)$. One where all four covariates entered both regression parts of the model, one where they only entered the survival part and one where they only entered the logistic part. Note that the second corresponds to treating the latent *Y*’s as independent and identically distributed, and the third to treating the diagnosis times of the susceptible group as independent and identically distributed. Estimates from a logistic regression on the event indicators *δ*, and from a Cox regression model (assuming no cured fraction), with the same four covariates, are included for comparative purposes. The nominal similarity of the estimates in the logistic model and Cox model to those of the logistic part of the cure models is discussed in Section 2.3.

**Figure 4.**
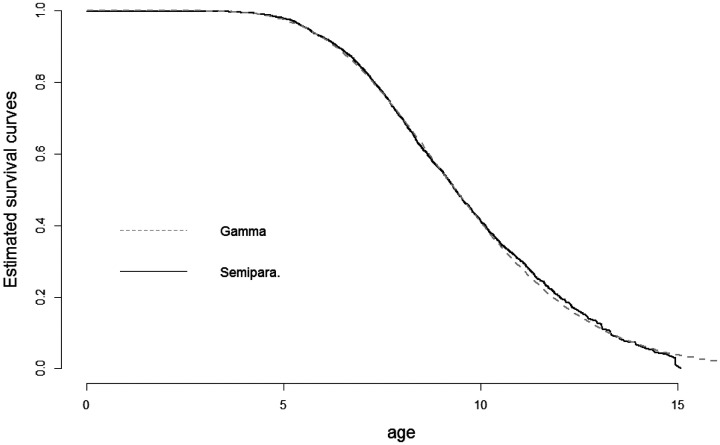
Estimates of the survival curve of the susceptible children, i.e. the proper survival functions *S*(*t*) in equation (5). The estimates are based on the model Semipara. 1 and Gamma 1 of Table 3.

Table 3 reports the parameter estimates and estimated standard errors of these for all eight models. Figure 4 displays estimates of the proper survival functions (that is, $S_{0}(t)$ in equation (5)) for the two cure models that treat the diagnosis times as independent and identically distributed (Gamma 1 and Semipara. 1).

**Table 3.** Estimates based on n=95 545 MoBa children.

	Logistic					Survival						
	*β*_0_	Paracetamol	Fever	Alcohol	Mother educ.	*a*	*b*	Paracetamol	Fever	Alcohol	Mother educ.	AIC
Logistic	–3.33 (−3.4, −3.26)	0.20 (0.11, 0.29)	0.18 (0.02, 0.33)	0.80 (0.19, 1.41)	–1.02 (−1.11, 0.94)							
Cox								0.19 (0.10, 0.28)	0.20 (0.05, 0.40)	0.76 (0.16, 1.35)	–0.92 (−1.00, −0.83)	
Gamma 1	–2.94 (−3.02, −2.86)	0.20 (0.11, 0.29)	0.21 (0.05, 0.37)	0.80 (0.17, 1.43)	–0.94 (−1.03, −0.85)	12.26 (11.27, 13.25)	1.27 (1.15, 1.40)					–28049.58
Gamma 2	–2.98 (−3.06, −2.89)	0.17 (0.05, 0.28)	0.45 (0.18, 0.72)	0.86 (0.03, 1.68)	–0.83 (−0.95, −0.71)	12.65 (11.56, 13.74)	1.34 (1.20, 1.49)	0.08 (−0.09, 0.24)	–0.47 (−0.84, −0.09)	–0.12 (−1.26, 1.10)	–0.23 (−0.41, −0.05)	–28041.23
Gamma 3	–2.90 (−2.98, −2.83)					10.91 (9.94, 11.89)	1.13 (1.00, 1.257)	0.23 (0.11, 0.35)	0.06 (−0.14, 0.12)	0.51 (−0.19, 1.20)	–1.30 (−1.42, −1.18)	–28139.40
Semipara. 1	–2.92 (−3.01, −2.83)	0.20 (0.11, 0.29)	0.21 (0.05, 0.37)	0.7 (0.04, 1.38)	–0.94 (−1.03, −0.85)							
Semipara. 2	–2.97 (−3.06, −2.88)	0.16 (−0.03, 0.29)	0.44 (0.22, 0.65)	0.79 (0.09, 1.49)	–0.83 (−0.96, −0.70)			0.07 (−0.11, 0.25)	–0.41 (−0.70, −0.12)	–0.05 (−0.73, 0.62)	–0.22 (−0.39, −0.05)	
Semipara. 3	–2.87 (−3.08, −2.66)							0.23 (0.10, 0.35)	0.08 (−0.11, 0.28)	0.46 (−0.19, 1.11)	–1.18 (−1.32, −1.04)	

Note: The semiparametric models were fitted using the smcure-package in R, with standard errors based on 100 bootstrap samples. The gamma density of the three parametric cure models is $\left(b^{a}/\Gamma (a))t^{a-1}\exp(-bt\right)$.

In Table 3, the first thing to notice is that in the cure models that include covariates on both the logistic and the survival part (Gamma 2 and Semipara. 2), the estimated effects of paracetamol on the logistic part are significant at the 95% level, while the estimated effects of paracetamol on the survival part is close to zero and insignificant at all reasonable significance levels. Among the three parametric cure models, Gamma 2 is the one with superior performance according to the Akaike information criterion (AIC), albeit only slightly better than the gamma model treating the diagnosis times as independent and identically distributed (Gamma 1). Not surprisingly, removing the paracetamol indicator and alcohol indicators from the survival part of the Gamma 2 model results in an improvement of the AIC score, that is, it has an AIC superior to all the models reported in Table 3 (this model has an AIC score of –28038.08. The estimates are similar and not reported).

The results reported in Table 3 are interesting because they can be seen as corroborating the reading of the paracetamol–ADHD hypothesis expounded in Section 2.1, namely that gestational exposure to paracetamol determines whether or not a child is susceptible, while being unimportant for the time to diagnosis. In other words, given susceptibility the time to diagnosis appears to be independent of the exposure.

The important issue of identifiability of the semiparametric cure model should be pointed out. Loosely speaking, for the fraction of susceptible children to be accurately estimated, we must assume that the (covariate dependent) distribution function of the survival times of the susceptible individuals reaches unity before the distribution function of the censoring times.^12^ In effect, for identifiability reasons, when we fit the semiparametric cure models, the survival functions are set to zero for all survival times above the largest observed diagnosis time. No such fix is demanded when fitting fully parametric cure models. See Section 4 for further discussion of these issues, and Amico and Van Keilegom^12^ for a thorough discussion of identifiability in semiparametric cure models.

## 4 Discussion and concluding remarks

In this section, we briefly discuss the above findings and introduce some topics for possible future research.

The cure model was motivated by arguing that the scientifically interesting question in many perinatal studies is how the exposure relates to a partly unobservable variable indicating whether or not the child is susceptible to the condition or disease of interest.

The empirical analysis of the paracetamol–ADHD hypothesis of Section 3 indicates that the diagnosis times are independent of the exposure when susceptibility is accounted for. These findings have important implications for studies on most childhood long-term outcomes as there will always be a fraction of the children that is never diagnosed with the condition studied, and among these many should, for all practical purposes, be regarded as nonsusceptible to the condition in question. When a fraction of the offspring are nonsusceptible, conventional survival analysis methods will give biased effect estimates.

### 4.1 Remark 1

As discussed in Section 2.1, when using the cure model, we assume that we do not have data on the absence of the condition or disease, i.e. *δ* = 0 does not inform us on what the true value of *Y* is. Now, consider a different scenario, where one does indeed have data on the absence of a condition or disease. Then, one would want to model a positive probability of nonsusceptibility (*Y *=* *0) being discovered. This motivates a model where the children born susceptible (*Y *=* *1) have a hazard rate α(t) governing the time to diagnosis, while the nonsusceptible (*Y *=* *0) children have a hazard rate β(t), governing the time to it is ascertained that they do not have the condition or disease under study. Define the variable, *D_i_* = *Y_i_* if $\delta_{i}=1$, and 0 otherwise. The likelihood function is then

$$L_{n}=\prod_{i=1}^{n}\;{\left(\pi_{i}\alpha (t_{i})e^{-\int_{0}^{t_{i}}a\left(s\right)\;\text{d}s}\right)}^{D_{i}\delta_{i}}{\left((1-\pi_{i})\beta \left(t_{i}\right)e^{-\int_{0}^{t_{i}}\beta \left(s\right)\;\text{d}s}\right)}^{(1-D_{i})\delta_{i}}\times {\left(\pi_{i}e^{-\int_{0}^{t_{i}}\alpha (s)\;\text{d}s}+(1-\pi_{i})e^{-\int_{0}^{t_{i}}\beta (s)\;\text{d}s}\right)}^{1-\delta_{i}}.$$

If *π*, α(t) and β(t) are parametrically specified, one can proceed with likelihood inference on this model. Theory for the situation where one or both of the hazard rates are nonparametric is a topic for further research.

### 4.2 Remark 2

A class of survival models that can give estimates of continuous levels of susceptibility are so called first hitting time models.^22,23^ One example is the following. Consider a Wiener process *Z*(*t*) with drift *μ* and $\text{Var}(Z(t))=\sigma^{2}$, starting at $c_{0}>0$. It is well known that the first time *Z*(*t*) hits zero follows an Inverse Gaussian distribution with parameters μ,σ and *c*_0_.^24^ Here, the parameter *c*_0_ can be interpreted as the degree of susceptibility, with higher values translating to lower degrees of susceptibility. One could also let *c*_0_ stem from some distribution on the positive half line and build some regression structure on this distribution. Moreover, if μ>0, then the distribution of the first hitting times is not proper. In particular, the probability of never being diagnosed is $1-\exp\{-2c_{0}\mu /\sigma^{2}\}>0$, which is what we want in order to allow for some of the children to be nonsusceptible to the condition in question.

### 4.3 Remark 3

We have argued that in the perinatal studies discussed in this paper, the quantity of scientific interest is *π*, the probability of being born susceptible, while parameters related to the distribution of the diagnosis times are nuisance parameters. Nevertheless, the model selection criterion employed in Table 3 is the AIC, a criterion that assesses general overall issues and goodness of fit aspects of the cure models, and not only how good the inference on *π* or related quantities is. Preferably, when the scientific question directs attention to one part of the cure model, the model selection criterion employed ought to reflect this. Therefore, a possible topic for future research is developing a focused information criterion (see Jullum and Hjort^25^ and Claeskens and Hjort^26^) for comparing different parametric, as well as parametric and semiparametric cure models. The idea is to select the model that best estimates a focus parameter, say *ψ*, where the quality of the estimator is assessed by (an estimate of) the mean squared error $\text{E}\;[{\left(\hat{\psi }-\psi \right)}^{2}]$. The obvious focus parameter in the context of the paracetamol–ADHD hypothesis is *β*_1_, but other interesting quantities include Pr⁡{susceptible|nondiagnosed at t}, or $\pi (x_{0}^{\text{t}}\beta )$, for a covariate vector *x*_0_ of particular interest.
